# Ultrastructural analysis of explanted CyPass microstents and correlation with clinical findings

**DOI:** 10.1007/s00417-022-05620-x

**Published:** 2022-03-09

**Authors:** Lisa Hübner, U. Schlötzer-Schrehardt, J. M. Weller, B. Hohberger, C. Y. Mardin, R. Lämmer

**Affiliations:** Department of Ophthalmology, Universitätsklinikum Erlangen, Friedrich-Alexander-Universität Erlangen-Nürnberg, Erlangen, Germany

**Keywords:** Glaucoma, MIGS, CyPass microstent, Fibrosis, Surgery failure

## Abstract

**Purpose:**

The purpose of this study was to obtain insight into cellular processes after CyPass microstent implantation into the supraciliary space. With this knowledge, we expected to find some reason for surgical failure.

**Methods:**

Nine CyPass microstents of 8 patients with primary open-angle glaucoma (*n* = 1), pseudoexfoliation glaucoma (*n* = 5), uveitic glaucoma (*n* = 1), and posttraumatic open-angle glaucoma (*n* = 1) were explanted due to recurrence of IOP elevation, corneal decompensation, or persistent hypotony. The explants were processed for light and transmission electron microscopy.

**Results:**

Fibrotic material, consisting of collagen fibrils, microfibrils, pseudoexfoliation fibrils produced by activated fibroblasts, was detected in the stent lumen of 4/5 pseudoexfoliation glaucoma patients and also in posttraumatic open-angle glaucoma. Fibrotic material was also present on the outer surface and within fenestrations of the majority of stents. Complete absence of fibrotic reaction was noticed in 3 of 9 microstents.

**Conclusion:**

Although MIGS is known to be less invasive than conventional surgery, implants placed in the suprachoroidal space may be adversely affected by a fibrotic tissue reaction resulting in implant failure. Understanding mechanisms and risk factors leading to fibrotic scarring following antiglaucomatous surgery may help to develop novel strategies that improve surgical outcome.


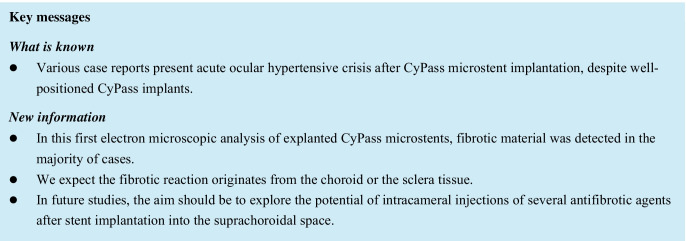


## Introduction

Glaucoma is a chronic, progressive optic neuropathy that represents the second leading cause of blindness globally [1]. It is defined by an acquired loss of retinal ganglion cells and axons within the optic nerve, astrocyte loss, as well as a corresponding progressive visual field damage.

Micro-invasive glaucoma surgery (MIGS) with implantation of stents has been developed to bridge the gap between medical therapy/laser treatment and conventional filtration surgery, which renders the best effect in intraocular pressure (IOP) reduction but carries also a higher risk for complications [2–4]. Even though MIGS procedures are less efficacious, they seem to be safer. Five criteria are known to describe MIGS: a micro-invasive approach, minimal tissue trauma, at least modest efficacy, rapid recovery, and a high safety profile [5]. Implantation of stents in order to improve aqueous outflow plays a significant role in MIGS procedures. Depending on type of stent, there are different approaches for IOP reduction: increasing trabecular outflow by bypassing the trabecular meshwork (iStent or Hydrus), increasing uveoscleral outflow via suprachoroidal pathways (CyPass microstent), or creating a subconjunctival drainage pathway (XEN gel stent) [6].

The CyPass microstent has received a CE mark from the European Union already in 2008. In July 2016, it was approved by the United States Food and Drug Administration [7]. This decision was based on the 2-year COMPASS trial results, showing sustained reduction of IOP along with the absence of vision-threatening microstent-related adverse events.

However, in 2018, Alcon declared voluntary withdrawal of the CyPass microstent due to the 5-year follow-up data collected in the COMPASS-XT trial that demonstrated sight-threatening adverse events associated with the CyPass implant [8]. Especially progressive corneal endothelial cell density loss (ECL) has been observed, which is caused by implant malposition.

Various case reports also presented acute ocular hypertensive crises after CyPass implantation although clinical examinations revealed deep and non-irritant anterior chambers as well as well-positioned implants [9, 10]. The authors assumed that delayed IOP elevation was caused by fibrosis in the suprachoroidal space, which may have resulted in a sudden elevation of IOP. Other causes suggested may be luminal occlusion, incorrect positioning, inflammation, and steroid sensitivity.

While the CyPass implant has been recalled in September 2018, lessons learned from its associated complications have to be explored for improved development of novel implants. Therefore, we report on a case series of CyPass microstents that had to be explanted due to IOP elevation, corneal decompensation, or persistent hypotony. Their histological and ultrastructural analysis provided insights into the pathological causes underlying surgical failure.

## Methods

In this retrospective, single-center, observational case series the histopathological findings of explanted CyPass microstents were investigated. Between 08/2017 and 08/2018, 233 CyPass microstents were implanted at the Department of Ophthalmology, University of Erlangen-Nürnberg, Germany by a single surgeon (RL). Out of these, 9 microstents of 8 patients were explanted between 01/2018 and 04/2020. The male/female ratio was 4/4, mean age at the time of implantation ranged from 48 to 80 years (66 ± 15 years). Patients suffered from primary open-angle glaucoma (*n* = 1), pseudoexfoliation glaucoma (*n* = 5), uveitic glaucoma (*n* = 1), and posttraumatic open-angle glaucoma (*n* = 1).

The CyPass microstent (Alcon, Fort Worth, TX, USA) is a fenestrated polyimide implant that improves uveoscleral outflow. The microstent is 8 mm in length, 430 µm in outer diameter, and 300 µm in inner diameter. Sixty-four fenestrations along the length of the stent allow circumferential outflow of aqueous into the suprachoroidal space. The microstent was inserted into the supraciliary space with a preloaded injector. Correct positioning could be validated by postoperative gonioscopy or anterior segment OCT, confirming that the proximal end rests in the anterior chamber angle.

Initial postoperative treatment after CyPass implantation was standardized in all patients, anti-inflammatory topical medication (prednisolonacetat 10 mg/ml or dexamethasone 1 mg/ml 5 times a day) was applied for 2 weeks, topical antibiotics (ofloxacin eye drops 3 mg/ml 3 times a day) for 1 week.

On the one hand, the decision for microstent explantation was made due to recurrence of IOP elevation refractory to medical treatment (*n* = 5). CyPass explantation then was performed in combination with further antiglaucomatous surgery. Even though the failure was not considered to be an indication for the stent removal, the microstent was removed to avoid any further intra- and/ or postoperative mechanical interaction between the shunt and the anterior chamber portion of the new drainage device.

Another reason for explantation was corneal decompensation (*n* = 3), which was defined by significant corneal edema in slit-lamp examination. Objective analysis consisted of corneal thickness measurement with Pentacam® HR (OCULUS Optikgeräte GmbH, Wetzlar, Germany) and reduction of visual acuity.

In one case, we had to decide for explantation due to persistent hypotony refractory to further surgery.

### Procedure for CyPass microstent removal

Surgical access for the microstent removal was ab-interno through clear cornea incisions. One paracentesis was constructed temporal before installing Z-HYALON plus (sodium hyaluronate 1.4%) into the anterior chamber. Afterwards, the gonioprism was used for visualization, then CyPass microstent was grasped with a 23G/0.6 mm Microforceps (Eckardt End-gripping, D.O.R.C., Düsseldorf, Germany) and removed gently towards the paracentesis. Finally, the chamber angle was visualized again by gonioprism, the remaining Z-HYALON plus was gently removed and the incisions were closed by hydrogenation.

After successful explantation, the CyPass microstents were fixed in 2.5% buffered glutaraldehyde and embedded in epoxy resin according to standard protocols. Semithin cross-Sects. (1 µm thick) were stained with toluidine blue. Ultrathin sections were stained with uranyl acetate and lead citrate and examined on a transmission electron microscope (EM 906E; Carl Zeiss Microscopy GmbH, Oberkochen, Germany).

The histopathological findings were correlated with clinical parameters such as glaucoma type, reason for intervention, and period of time until explantation.

## Results

### Patients’ characteristics

Types of glaucoma were pseudoexfoliation glaucoma (PXG) in 5 cases, as well as posttraumatic OAG, uveitic glaucoma (UVG), pigmentary glaucoma (PDG), and POAG one each (Table 1). Elevated IOP refractory to medical treatment was the reason for CyPass microstent explantation in 5 eyes (posttraumatic OAG, PDG, and 3 of PXG), corneal decompensation in three eyes (PXG, UVG, POAG), and refractory hypotony in one eye (PXG). In the latter, interventions like intraluminal suture occlusion or repeated pneumoretinopexie did not lead to improvement. Clinical examinations revealed significant choroidal folds at the posterior pole along with a considerable reduction of visual acuity (20/30 before CyPass implantation compared with 20/200 nearly 2 months post-surgery). The IOP before explantation was 6 mmHg.

**Table 1:** Patients’ characteristics

No	Gender, age	Systemic diseases	Type of glaucoma	Previous surgeries	Therapy before impl.	Time till expl.	Reason for expl.	Max. IOP before impl. [mmhg]	Therapy after impl.	IOP before expl. [mmhg]	Corneal thickness before impl. [µm]	Corneal thickness before expl. [µm]
1	m, 80y	Sleep apnea syndrome, atrial fibrillation, hypertension	PXG	cataract surgery	PG-analog CA-inhibitor β-blocker (preserved)	1 mo	IOP elevation	20	Dexamethasone 3 × /day	28	−	−
2	m, 48y	None	PXG	CPC	PG-analog, CA-inhibitor β-blocker (unpreserved)	1 mo	IOP elevation	25	Dexamethasone 5 × /day	40	−	−
3	m, 48y	None	PXG	CPC	PG-analog sympatho-mimetic agent (preserved)	23 mo	corneal decompensation	40	Prednisolon 5 × /day	16	746	1138
4	f, 80y	Thyroid disease, cardiac pacemaker	PXG	cataract surgery XEN	PG-analog CA-inhibitor β-blocker (unpreserved)	1 mo	IOP elevation	32	Dexamethasone 3 × /day	47	−	−
5	f, 63y	Breast cancer	PXG	none	PG-analog CA-inhibitor β-blocker α2-agonist (preserved)	1 mo	Refractory hypotony	23	Prednisolon 5 × /day	4	−	−
6	m, 58y	Diabetes mellitus, hypertension	OAG posttraumatic	SLT, CPC, TE, XEN, bleb needling	PG-analog CA-inhibitor β-blocker α2-agonist (preserved)	4 mo	IOP elevation	46	Dexamethasone 5 × /day	31	−	−
7	f, 80y	Hypertension, Atrial fibrillation thyroid disease	UVG	CPC, cataract surgery	PG-analog CA-inhibitor β-blocker α2-agonist (unpreserved)	24 mo	Corneal decompensation	29	Prednisolon 3 × /day	14	834	1030
8	m, 51y	Thyroid disease, COPD	PDG	SLT, XEN, bleb needling	CA-inhibitor β-blocker (preserverd)	22 mo	IOP elevation	42	Dexamethasone 3 × /day	37	−	−
9	f, 70y	None	POAG	XEN	PG-analog β-blocker (unpreserved)	26 mo	Corneal decompensation	38	Dexamethasone 3 × /day	11	608	797

*m*, male; *f*, female; *CPC*, cyclophotocoagulation; *IOP*, intraocular pressure; *OAG*, open-angle glaucoma; *PDG*, pigmentary glaucoma;* POAG*, primary open-angle glaucoma; *PXG*, pseudoexfoliation glaucoma; *SLT*, selective laser trabeculoplasty; *TE*, trabeculectomy; *UVG*, uveitic glaucoma; *dexa*, dexamethasone; *predni*, prednisoloneacetat; *CA-inhibitor*, carbonic anhydrase inhibitor; *PG-analog*, prostaglandin analogue

The interval between implantation and explantation ranged from 1 to 26 months (12 ± 12 months). Explantations due to recurrence of IOP elevation or hypotony were performed after 1 to 22 months, all of them after a shorter interval than those caused by corneal decompensation (23 to 26 months).

Eight eyes underwent previous surgery before CyPass implantation (selective laser trabeculoplasty, transscleral cyclophotocoagulation, trabeculectomy, XEN gel stent implantation, bleb needling).

**Fig. 1:**
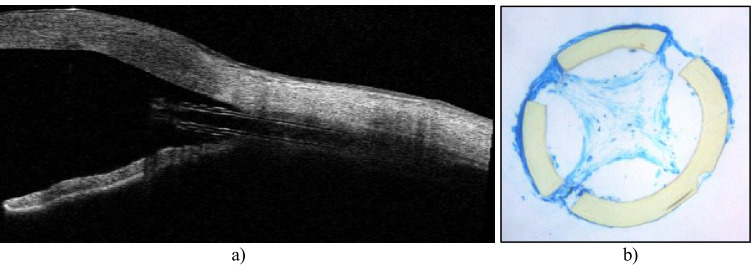
**a** Anterior segment optical coherence tomography (Cassia II, Tomey, Nagoya, Japan). 4 weeks after CyPass microstent implantation. Intraocular pressure was 38 mmHg. We miss accumulation of aqueous around and posterior to the microstent. Instead, one can see hyperreflective tissue inside the lumen and on the external surface. **b** Corresponding light microscopic analysis after stent explantation revealed marked fibrotic material within the stent lumen and also surrounding the microstent

Mean IOP before CyPass explantation due to recurrence of IOP elevation was 37 mmHg (± 7 mmHg). In gonioscopy, the microstents were well positioned with 1 retention ring in the anterior chamber. Figure 1 shows a corresponding anterior segment optical coherence tomography of patient no. 2 (Casia II, Tomey, Nagoya, Japan).

Decision for CyPass removal due to corneal decompensation was made depending on increase of corneal thickness in combination with slit-lamp examination. In gonioscopy, the microstents were well positioned with 1 retention ring in anterior chamber. From time before CyPass implantation to explantation, the increase of corneal thickness was 392 µm (no. 3), 196 µm (no. 7), and 189 µm (no. 9). In accordance, we noticed loss in vision: HM to LP (no. 3), 20/40 to 20/400 (no. 7), 20/200 to FC (no. 9).

### Histopathology

**Fig. 2:**
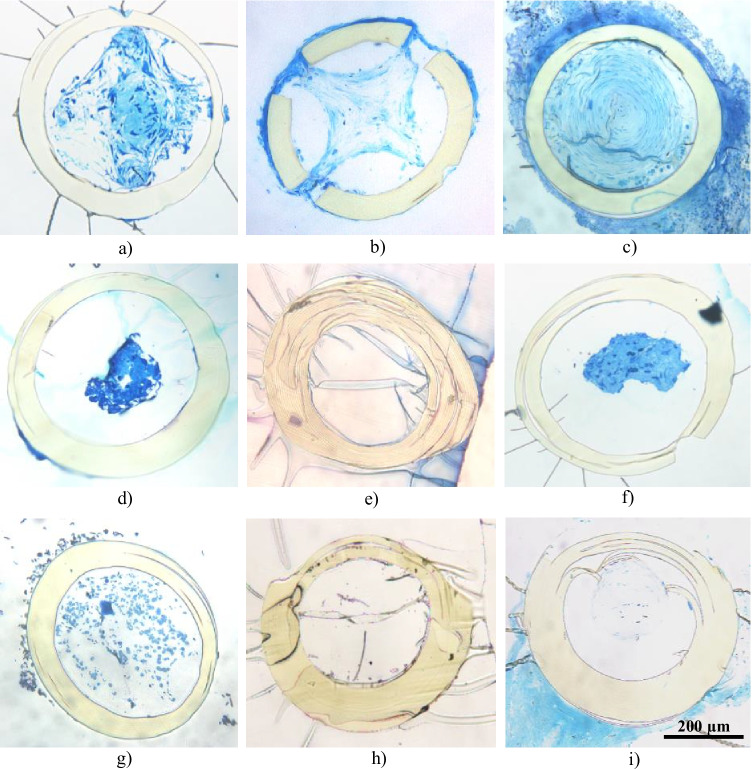
Light microscopy analysis (toluidine blue staining). **a**, **b**, **c**, **d** Pseudoexfoliation glaucoma. Marked fibrosis within stent lumen (nos. 1–4). In **b**, **c**,**d**, **i**, fibrotic material also on external surface. **e** Pseudoexfoliation glaucoma. Lumen without any substance (no. 5). **f** Posttraumatic open-angle glaucoma. Fibrosis in lumen (no. 6). **g** Uveitic glaucoma, no fibrotic material in lumen or on external surface (no. 7). **h** Pigmentary glaucoma. Lumen without any substance (no. 8). **i** Primary open-angle glaucoma. Fibrosis on external surface with collagen, lumen without any substance (no. 9)

**Fig. 3:**
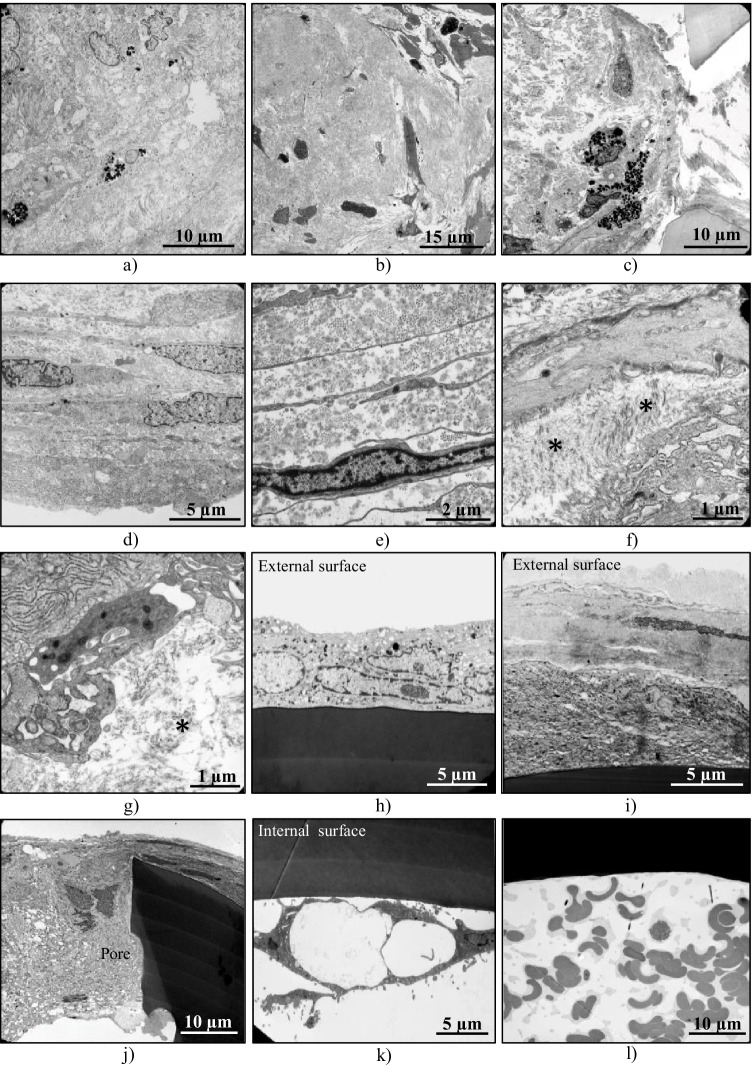
Transmission electron microscopic analysis. **a**,**b**, **c**, **d, e** Intraluminal fibrotic deposits, composed of activated fibroblast-like cells embedded within a fibrous extracellular matrix consisting of collagen fibrils and microfibrils (nos. 1–5). **f**,**g** Deposits of pseudoexfoliation material in PXG specimens (*) (nos. 1, 4). **h**, **i** The fibrotic material covering the outer stent surface contained both cellular and extracellular fibrous components (no. 4, 2). **j** The surface cells were seen to migrate through the fenestrations and coat the inner stent surface (no. 2). **k** Scattered fibroblastic cells along the inner wall (no. 5). **l** In the UVG specimen, only erythrocytes were found within the stent lumen (no. 7)

Light microscopic analysis of semithin cross-sections showed different amounts of extracellular material accumulation in the lumen of the stents (Table 2, Fig. 2). Fibrotic material was detected in the stent lumen of 4/5 PXG cases as well as in posttraumatic OAG, whereas empty lumina were found in one PXG patient suffering from persistent hypotony as well as in PDG and POAG specimens. In uveitic glaucoma, the stent lumen contained blood cells and fibrin. Fibrotic material covering the external surface of the stent was present in 3 PXG and in POAG specimens. Occasionally, fibrotic material was found within the fenestrations of the stent wall, indicating ingrowth from the outer surface (Fig. 3k). There were no signs of inflammatory cells on the surface or in the lumen of the stents.

**Table 2:** Overview of findings obtained from microscopic analysis

No	Type of glaucoma	Time till explantation	Reason for explantation	Histopathological findings
1	PXG	1 mo.	IOP elevation	Lumen with pronounced fibrosis • Fibroblast-like cells, pigmented cells, large amounts of extracellular matrix • Focal deposits of pseudoexfoliation material
2	PXG	1 mo.	IOP elevation	Lumen with pronounced fibrosis • Fibroblast-like cells, large amounts of extracellular matrix, pigmented cells • Marked fibrosis on external surface • Surface cells seem to migrate through the fenestration and coat the inner stent surface
3	PXG	23 mo.	Corneal decompensation	Pronounced fibrosis (lumen completely obstructed) • Fibroblast-like cells and collagenous extracellular matrix forming concentric layers • Marked fibrosis on external surface
4	PXG	1 mo.	IOP elevation	Lumen with moderate fibrosis • Foci of pseudoexfoliation fibrils • Cell sheath on outer surface
5	PXG	1 mo.	Refractory hypotony	lumen empty, but scattered fibroblastic cells along the inner wall
6	OAG posttraumatic	4 mo.	IOP elevation	Lumen with moderate fibrosis • Fibroblast-like cells, collagen fibers
7	UVG	24 mo.	Corneal decompensation	Lumen without fibrosis • Erythrocytes, few leukocytes and fibrin within lumen and on outer surface
8	PDG	22 mo.	IOP elevation	Lumen empty
9	POAG	26 mo.	Corneal decompensation	Lumen empty • Marked fibrosis on external surface

*IOP*, intraocular pressure; *OAG*, open-angle glaucoma; *PDG*, pigmentary glaucoma;* POAG*, primary open-angle glaucoma; *PXG*, pseudoexfoliation glaucoma;* UVG*, uveitic glaucoma

Transmission electron microscopy revealed that the intraluminal fibrotic deposits were composed of activated fibroblast-like cells embedded within a fibrous extracellular matrix consisting of collagen fibrils and microfibrils (Fig. 3a–e). Occasionally, pigmented cells could be also observed (Fig. 3a,c). In all PXG specimens, there was evidence of focal production of typical pseudoexfoliation fibrils by activated fibroblasts (Fig. 3f,g). Similarly, the fibrotic material covering the outer stent surface contained both cellular and extracellular fibrous components (Fig. 3h,i). At intervals, the surface cells were seen to migrate through the fenestrations and coat the inner stent surface (Fig. 3j). In the UVG specimen, only erythrocytes were found within the stent lumen (Fig. 3l).

We did not find any correlation between previous surgery and fibrotic reaction. Regarding the only eye without any previous surgery, there did not exist fibrotic material inside the stent lumen but fibroblastic cells along the internal surface were present. There also was no difference apparent with regard to post-surgery treatment, whether prednisolone was applied or dexamethasone.

In addition, we did not find any correlation between development of fibrosis and recurrence of IOP elevation before explantation. Microstent analysis of one eye with marked IOP elevation (37 mmHg) preoperatively did not show any fibrosis, whereas another eye (16 mmHg) suffered from fibrotic reaction.

Furthermore, we could not identify any correlation between underlying systemic diseases and the occurrence of fibrosis.

## Discussion

We present 9 cases of explanted CyPass microstents and the corresponding histopathological analysis in order to obtain a better understanding of implant failure. The stents were initially implanted in patients with medically refractive glaucoma. Explantations were performed due to recurrence of IOP elevation, persistent hypotony, or corneal decompensation. Electron microscopic analysis revealed fibrotic material not only on the external surface of microstents but also within their lumen and, occasionally, within fenestrations of the stent wall. Therefore, it can be assumed that cells producing fibrotic material transverse the fenestrations. Although MIGS is known to be less invasive than conventional surgery, implants placed in the suprachoroidal space apparently are not safe from tissue reaction. As a result, implant failure due to fibrosis can occur. It is important to understand mechanisms and risk factors for scar formation subsequent to antiglaucomatous surgery. This may offer the opportunity to develop strategies that improve surgical outcome.

In our case series, more than half of the explanted microstents were from eyes with PXG. Moderate to pronounced fibrosis was evident in the majority (4/5) of PXG specimens. PXG is known to be the most common type of secondary OAG [11]. This type of glaucoma is more aggressive than POAG due to higher levels and greater fluctuations of IOP. Although the reason for an increased risk of fibrosis after suprachoroidal stent implantation in PXG eyes has not been elucidated, it may be related to the fibrotic nature of the underlying disease, pseudoexfoliaton syndrome (PXS). PXS has been described as a fibrotic process characterized by the excessive production of abnormal fibrillar aggregates, which progressively accumulate throughout the anterior eye segment and connective tissues of various organ systems and blood vessel walls [12]. Abnormal fibrotic PXS deposits have been observed throughout all tissues of the aqueous humor outflow pathways, including trabecular meshwork, periphery of Schlemm’s canal, collector channels, and aqueous veins, as well as in the suprachoroidal space [13]. Thus, it may be assumed that eyes with PXG are more prone to fibrotic alterations of suprachoroidal glaucoma implants. However, Gürlü et al. [14] found no difference in the success of trabeculectomy between PXG and POAG, as well as no difference in efficacy of XEN gel stent implantation between PXG and PDG [15–17].

In one PXG eye, microscopic analysis did not reveal any fibrotic material in the device lumen. This was the only eye that did not experience any ophthalmological surgeries before the CyPass implantation. Post-surgery, the patient was suffering from symptomatic hypotony refractory to further surgeries (intraluminal suture occlusion, repeated pneumoretinopexie). Although we did not see any intraluminal material in microscopic analysis, there were scattered fibroblastic cells along the inner wall of the microstent, which may be indicative of an early stage of intraluminal invasion and subsequent fibrosis. It cannot be ruled out that the normal wound healing in this patient did not work well, so that there effectively was not observed any relevant fibrotic reaction.

No fibrotic material was found in the CyPass stent explanted from uveitic glaucoma. This seems to be interesting as Qureshi [18] reported about even lower needling rates in uveitic patients compared to POAG after XEN gel stent implantation.

Appearance of fibrosis was independent of the interval between implantation and explantation. On the one hand, we observed marked fibrosis in microstents that were explanted just 1 month after implantation. On the other hand, there were microstents explanted after more than 20 months that did not reveal any fibrotic material.

Mariotti et al. [19] were able to demonstrate the presence of a diabetic pre-existing condition with an increased risk of failure after EX-PRESS shunt implantation. In the patient group presented here, there was only one patient with diabetes mellitus, so we cannot make any statements in this regard. We found moderate fibrosis in this patient.

Consistent with our experiences, various case reports present acute ocular hypertensive crises after stent implantation, whereas clinical examinations revealed deep and non-irritant anterior chambers as well as well-positioned CyPass implants [9, 10]. The authors assumed that delayed IOP elevation was caused by fibrosis of the suprachoroidal space, which may have resulted in a sudden elevation of intraocular pressure. Other causes may be luminal occlusion, incorrect positioning, inflammation, and steroid sensitivity.

Fibrosis is known to be a pathological wound healing where an excessive accumulation of extracellular matrix components results. Increased angiogenesis and migration of fibroblasts lead to fibroblast proliferation, followed by collagen deposition. Surgery itself causes expression of growth factors that in the end stimulates production of collagen [20].

Until now, studies focused on investigation of fibrotic reaction after conventional glaucoma surgery. It has been shown that scar formation after filtering surgery may lead to surgery failure by restriction of aqueous flow across the filtering bleb [21]. Modulation of normal wound healing by antifibrotic agents is supposed to improve surgical success. Studies have shown that Tenon’s capsule fibroblasts (HTFs), which are located beneath the conjunctiva, play a major part in this pathway of scarring. Therefore, we have to distinguish molecular mechanisms following filtration surgery from those following CyPass microstent implantation. We do not expect the fibrotic reaction originate from the conjunctiva or Tenon’s capsule after creating a shunt from the anterior chamber to the suprachoroidal space, but more likely from the choroid and/ or the sclera tissue (both bordering the suprachoroidal space). Löber et al. [22] investigated mRNA profiles of fibroblasts from the choroid, sclera, and Tenon’s space to get information about potential pharmacological targets for fibrosis prevention. Common to all three cell types was the presence of components that belong to the transforming growth factor beta (TGFβ) signaling pathway. Therefore, targeting the TGFβ pathway, which is also activated in tissues of POAG and PXS/PXG patients [13], seems to be a valuable objective for preventing fibrotic reaction [23, 24].

Pitha et al. [25] evaluated prevention of TGFβ-induced transdifferentiation of cultured scleral fibroblasts to myofibroblasts by Rho-associated protein kinase (ROCK) inhibitors and its potential to reduce fibroblast proliferation in response to chronic IOP elevation. ROCK inhibitors H1152, Y27632, and fasudil-reduced smooth muscle actin (SMA) expression (68 to 85%) and collagen gel contraction (27 to 36%) by scleral fibroblasts. The Rho-kinase inhibitor Ripasudil also demonstrated IOP-lowering effect in PDG by reducing trabecular meshwork stress fibers and restored migration of trabecular meshwork cells (Wang et al. 2020) [26].

Inhibition of vascular endothelial growth factor (VEGF) is described in a few studies with regard to prevent fibrotic reaction after filtering surgery. VEGF expression was proven in Tenon tissue following filtering surgery, where VEGF induces proliferation of Tenon’s fibroblasts [27]. It therefore has been assumed to represent a link between angiogenesis and scar formation after filtrating surgery [28]. Only limited data are avaiable regarding the role of VEGF in wound healing following shunt implantation into the suprachoroidal space. Hoeh et al. [29] demonstrated positive effects of bevacizumab into the anterior chamber postoperative to CyPass implantation. They named it a safe intervention, which reduces iris tissue reactions and improves efficacy up to 6 months postoperatively. Clinically, effect of IOP lowering was noticed, even if the extent was limited. However, they focused on clinical effect of bevacizumab intracameral, but they did not investigate the occurrence of fibrosis with and without bevacizumab.

In general, data of explanted suprachoroidal shunts are rare. Agnifili et al. [30] reported about light microscopic analysis of failed gold microshunts in primary open-angle glaucoma in five eyes. The main findings were the presence of connective tissue filling the inner spaces and creating a thick fibrotic capsule surrounding the ends of the device. However, they did not perform electron microscopic analysis. Berk et al. [31] also performed removal of a gold microshunt.in POAG. Light and electron microscopic analysis revealed images of a fibrotic pseudo-capsule traversing its microchannels and fenestrations.

### Limitations

In this study, we reviewed only patients with Cypass microstents that had to be explanted because of complications. Therefore, we only have limited information about successful IOP-lowering effect after Cypass implantations. Three eyes that suffered from corneal decompensation have to be classified as successful IOP-lowering stents. It is notable that microscopic analysis of one of them revealed remarkable fibrosis inside the lumen, but IOP decreased from 40 mmHg before stent implantation to 16 mmHg before removal.

Furthermore, significance is restricted due to small number of cases. However, it is still the largest case series of explanted CyPass stents which were analyzed by electron microscopy. We were able to demonstrate the occurrence of fibrosis within and around the CyPass stent, but we are not finally able to comprehend the main reasons for such complications.

Regarding microstents without any substance within the lumen, we cannot exclude that existing fibrotic material was pulled out during microstent explantation.

### Future perspectives

Until now, the potential of intracameral drug injection after stent implantation is not much investigated. Only Hoeh et al. [29] published a study about IOP-lowering effect of bevacizumab into the anterior chamber after CyPass microstent implantation, whereby molecular mechanisms were not investigated.

Another approach for the control of wound healing could be the use of glucocorticoids, like it is used after bleb surgery. However, there is no knowledge about the effects on scleral and choroidal fibroblasts. In future studies, the aim should be to explore the potential of intracameral injections of several antifibrotic agents after stent implantation, including VEGF antibodies and glucocorticoids.

## Conclusions

Even though MIGS is supposed to be less invasive than filtrating surgery, we have learned that even suprachoroidal devices still carry the risk of fibrosis similar to filtrating surgery. We represent the first electron microscopic analysis of explanted Cypass microstents providing evidence of fibrotic material within the stent lumen and on the external surface. However, there is only limited data about wound healing and fibrosis in the suprachoroidal space. Although the Cypass microstent was already withdrawn from the market, it is important to learn from its complications to improve development of novel implants.
